# Hybrid complexes of high and low molecular weight hyaluronan delay in vitro replicative senescence of mesenchymal stromal cells: a pilot study for future therapeutic application

**DOI:** 10.18632/aging.101493

**Published:** 2018-07-12

**Authors:** Nicola Alessio, Antonietta Stellavato, Tiziana Squillaro, Stefania Del Gaudio, Giovanni Di Bernardo, Gianfranco Peluso, Mario De Rosa, Chiara Schiraldi, Umberto Galderisi

**Affiliations:** 1 Sbarro Institute for Cancer Research and Molecular Medicine, Center for Biotechnology, Temple University, Philadelphia, PA 19107, USA; 2 Department of Experimental Medicine, Biotechnology and Molecular Biology Section, University of Campania “Luigi Vanvitelli” , Naples, Italy; 3 Institute of Agri-Environmental Biology and Forestry (IBAF), CNR, Naples, Italy

**Keywords:** mesenchymal stromal cells, hyaluronan, senescence, extracellular matrix

## Abstract

Mesenchymal stem cells, a subpopulation of mesenchymal stromal cells (MSCs), are present in the stroma of several tissues. MSC *in vitro* cultivation for clinical treatments may greatly affect MSC properties. A primary handicap is replicative senescence that impairs MSC functions. Hyaluronan (HA) is present in the extracellular matrix that composes the stem cell niche environment and is under investigation as a key factor for *in vitro* stem cell growth. We evaluated the effect on MSC cultivation of HA hybrid cooperative complexes (HCC) that are obtained from high (H) and low (L) weight molecules (NAHYCO™). We compared this HCC with H-HA and L-HA. We investigated the effects of these HAs on proliferation, cell cycle, apoptosis, senescence, and differentiation following the addition of the polymer solutions in the culture media at concentrations that did not drastically modify the medium viscosity. Interestingly, 0,16% HCC significantly delayed the senescence compared with the controls. This occurred without alteration of the cell cycle, cytotoxicity, or apoptosis. HCCs also promoted adipogenic and chondrogenic differentiation. Our finding could suggest a potential functional role of HCC above the updated scientific reports of its effects and pave the way to optimization of MSC cultivation for therapeutic application.

## Introduction

Mesenchymal stem cells are a subpopulation of mesenchymal stromal cells (MSCs) that are present in the stroma component of several organs and tissues, mainly in bone marrow and adipose tissue [1].

According to the International Society for Cellular Therapy (ISCT) criteria, the isolation of MSCs produces heterogeneous, non-clonal cultures of stromal cells containing stem cells with different multipotential properties, committed progenitors, and differentiated cells. The stem cells present in MSC cultures can differentiate into osteocytes, chondrocytes, adipocytes, and smooth muscle cells. MSCs also present immunomodulatory properties and the capacity to sustain tissue repair and homeostasis. All these features have paved the way to several potential therapeutic applications of MSCs [2].

*In vitro* cultivation is a necessary procedure to obtain an elevated number of MSCs for clinical purposes. This poses fundamental issues since *in vitro* expansion of MSCs may greatly affect their properties. A major obstacle is the onset of replicative senescence. Senescent cells are non-functional cells that may negatively influence the activity of surrounding healthy cells by releasing several paracrine factors [3]. Therefore, the presence of senescent cells has to be carefully evaluated in order to safeguard the therapeutic potential of any MSC batch destined for patients.

Several studies have demonstrated that *in vitro* cultivation procedures should mimic the physiological environment in order to obtain functional cells. Indeed, the interactions of MSCs with their microenvironment play an important role in their morphogenesis and downstream differentiation commitment. These interactions are partially mediated by cell surface receptors and extracellular matrix (ECM) components. A starting point to find new strategies in treating debilitating diseases and tissue damage may be a grow procedure based on manipulation of ECM components that could induce MSC growth and/or differentiation without the onset of senescence.

In this setting, we focused our attention on hyaluronan (also known as hyaluronate or hyaluronic acid, HA), which is a key component of many extracellular matrices, including those forming the stem cell environment. HA is a nonsulfated, linear polysaccharide with unique physical and mechanical properties. It contributes to the maintenance of tissue hydration and modulates solute diffusion through the extracellular space; moreover, HA shows important functional and mechanical properties (e.g., in joint lubrication). HA interacts with various proteins or proteoglycans in the ECM, on the cell surface, and within the cytosol. The biological functions of HA depend on (a) its molecular weight, (b) the HA binding proteins, (c) its spatial and temporal distribution in tissues, and (d) the cellular background and tissue stages. Roles of HA include cartilage matrix stabilization, angiogenesis, cell mobility, inflammation regulation, and growth factor action [4,5].

Taking advantage of the inherent biocompatibility and biodegradability of HA as well as its susceptibility to chemical modification, researchers have developed various HA-based biomaterials and tissue constructs with promising and broad clinical potential.

Due to its abundance in the stem cell niche, we evaluated the effect on MSC cultivation of HA hybrid complexes (HCC) that are obtained from high (H) and low (L) weight molecules (NAHYCO™ technology). The importance of the use of HCC with respect to linear HA is related to the *in vitro* approaches, in that these compounds could improve keratinocytes wound reparation [6] and significantly increase collagens and elastin production in keratinocytes and fibroblasts as well in a 3D skin model [7]. Also, the injection of HCC in the subdermal fat compartment may recruit and differentiate stem cells in adipocytes in order to induce fat tissue restoration [8].

## RESULTS

While there are many surface markers to isolate MSCs, current protocols are based on differential attachment properties of MSCs with respect to other mononuclear cells present in bone marrow aspirates. This procedure is also currently used for “medical grade” MSCs that must be positive for surface markers (CD73, CD90, and CD105) [9]. Monocytes and macrophages are the most common contaminant present in primary MSC cultures. Their presence strongly decreases starting from culture passage 2 [10]. Any attachment factor, such as hyaluronan, may interfere with a process aiming to reduce macrophage contamination. For this reason, all treatments on MSC cultures started at passages 2-3. At the beginning of each experiment, we evaluated that MSC cultures fulfill the ISCT (International Society for Cellular Therapy) criteria and were devoid of monocytes/macrophages (data not shown) [9].

We tested three different HA formulations on MSC cultures: (a) 120±20 kDa or low molecular weight HA (L-HA); (b) 1400±200 kDa or high molecular weight HA (H-HA); and (c) hybrid cooperative complexes of low and high molecular weight (HCC) obtained through the patented NAHYCO™ technology. MSCs were incubated with either 0.16% or 0.5% of each HA formulation. We previously demonstrated that these concentrations did not negatively affect cell functions and vitality [8]. The NAHYCO technology is based on thermal cross-linking. This changes the HA behavior resulting in the formation of co-operative hybrid complexes. This is also the explanation for the different biological behavior with respect to H-HA and L-HA alone. One of the main advantages is longevity. Hybrid cooperative complexes have proven to be very stable with natural hyaluronidase (BTH) digestion when compared to H-HA [7]

### Effects of HA formulations on cell cycle, apoptosis, and senescence

We incubated MSCs with media containing the different HA formulations for one, two, three and seven days. On these cells, we performed several biological assays. Here we present data following three days of treatment. These results overlapped those obtained at the other time points (data not shown).

Ki67 immunostaining is currently used to detect the percentage of cycling (G_1_; S; G_2_/M) and resting cells (G_0_) that are Ki67 positive and negative, respectively. Nevertheless, cycling cells must have high translational levels in order to produce enough proteins for cell growth and proliferation. A key protein involved in the regulation of protein translation is the ribosomal protein S6 (RPS6), a component of the 40S ribosomal subunit. In cells with active protein production, RPS6 is phosphorylated via the mTOR pathway. The level of phosphorylated RPS6 (pRPS6) is elevated in cycling cells and is low or negligible in resting (G_0_) cells. The combination of these two proteins allows the identification of cycling cells (Ki67(+) pRPS6(+) and quiescent cells (Ki67(-) pRPS6(-)) [11,12].

**Figure 1:**
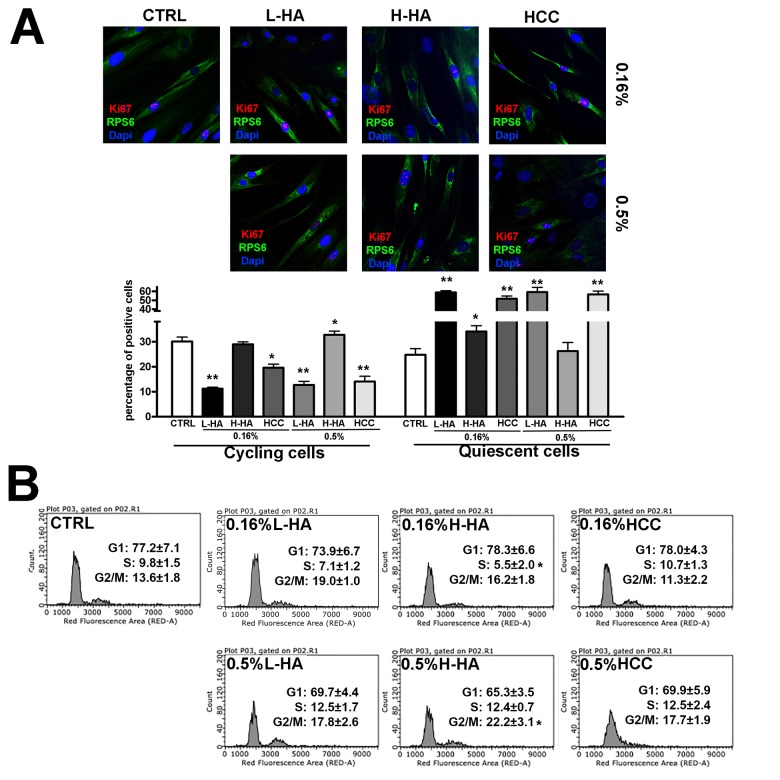
(**A**) Representative microscopic field of Ki67 immunostaining (red) and pRPS6 (green) on MSCs grown in the presence of different hyaluronan mixtures. Nuclei were counterstained with Hoechst 33342 (blue). The graph shows the percentage of Ki67(+) pRPS6(+) cycling cells and Ki67(-) pRPS6(-) quiescent cells. Data are expressed with standard deviation (n = 3, *p<0.05, **p<0.01). (**B**) The picture shows representative FACS analysis of MSCs grown in the presence of different hyaluronan mixtures. Experiments were conducted in triplicate for each condition. Percentages of different cell populations (G_1_, S, and G_2_/M) are indicated. Data are expressed with standard deviation (n = 3, *p<0.05).

We evaluated the effect of the several HA formulations on MSC cultures by determining the Ki67 pRPS6 immunostaining in every tested condition. Cells were incubated for three days in media supplemented with the different HA formulations. In this way, we identified the actual proportion of cycling cells (Fig. 1A).

MSCs cultivated in L-HA either at 0.16% or 0.5% concentration showed a significant reduction in cycling cells compared with the controls, and this was accompanied by a concomitant increase in quiescent cells (Fig. 1A). No substantial modifications in the number of cycling and resting cells occurred in cultures treated with H-HA (Fig. 1A). Cells incubated with 0.16% or 0.5% of HCC showed a decline in the percentage of cycling cells and an increment of resting cells (Fig. 1A).

Flow cytometry analysis of MSC cultures evidenced that all the experimental conditions, but 0.16% HCC, modified the pattern of cell cycle profiles with respect to control conditions (Fig. 1B). For example, we detected a general increase in the percentage of G_2_/M cells and reduction of S phase cells in 0.16% H-HA. The observed changes were not statistically significant; nevertheless, this may suggest that HA treatments may partially alter the *in vitro* MSC cycling process.

**Figure 2:**
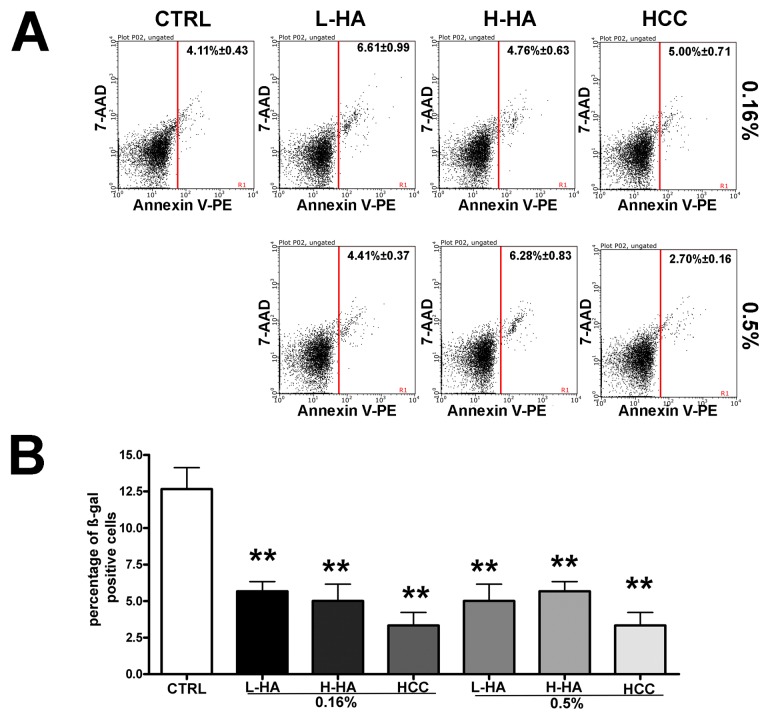
(**A**) Flow cytometry analysis of apoptosis with Annexin assay. The assay allows the identification of early (Annexin V + and 7ADD -) and late apoptosis (Annexin V + and 7ADD +). Nevertheless, apoptosis is a continuous process, and we calculated the percentage of apoptosis as the sum of early and late apoptotic cells. For every experimental condition, the percentage of apoptotic cells is indicated in the upper right corner of the analysis plot. Data are expressed with standard deviation (n = 3, *p<0.05). (**B**) Acid beta-galactosidase Senescence assay. The graph shows the mean percentage value of senescent cells in every experimental condition (± SD, n = 3, *p<0.05).

The apoptosis process was not affected by incubation with the different HA mixtures (Fig. 2A) while senescence was noticeably reduced (Fig. 2B), as detected by annexin V flow cytometry analysis and *in situ* beta-galactosidase assay, respectively. In particular, HCC treatment reduced more than three times the percentage of senescent cells. Ki67 and pRPS6 immunostaining confirmed the decline in senescence. Senescent cells have a high metabolic activity, are permanently arrested, and hence are Ki67(-) pRPS6(+). Indeed, in HCC treated cells, we observed a decline of Ki67(-) pRPS6(+) cells compared with the controls. In detail, in cells incubated with 0.16% HCC, we detected 18.4% Ki67(-) pRPS6(+) cells while in the control culture this value was 31.1%. Also, the treatment with 0.5% HCC reduced the number of Ki67(-) pRPS6(+) cells (20.4% versus 31.1%). It must be underlined that leaving the cell cycle and entering quiescence is a progressive process. For this reason, within a Ki67(-) pRPS6(+) cell population, some cells are true senescent cells (i.e., cell cycle arrested cells with a very active metabolism), others are becoming quiescent and still have a residual pRPS6 staining. Nevertheless, reduction of this cell population lends further credit to the beta-galactosidase data showing a decrease in senescence following treatment of MSCs with the HCC mixture.

We then evaluated the long-term effects of 0.16% HCC on MSCs since this was the experimental condition with the best biological performance (no alteration in cell cycle profile, no change in cell death percentage, and a huge reduction in senescence). Indeed, after 30 days of incubation in 0.16% HCC, the percentage of senescent cells was still low (9.0% +/- 1.6%) compared with controls (39.1% +/- 2.8%).

### Stemness and differentiation potential

**Figure 3:**
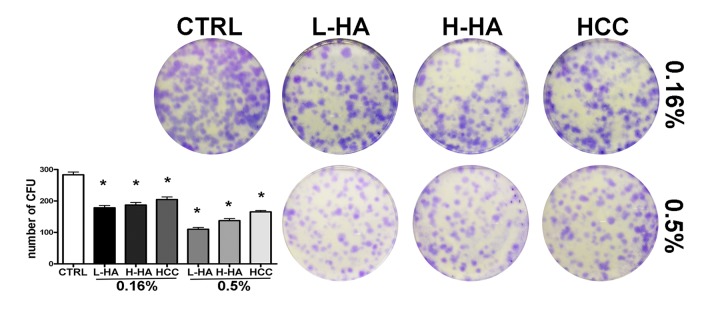
CFU assay. The pictures show representative crystal violet staining of clones obtained after 14 days of incubation with MSCs plated following treatment with different HA solutions. The mean number of clones (± SD, n = 3, *p<0.05) is indicated in the histogram.

**Figure 4:**
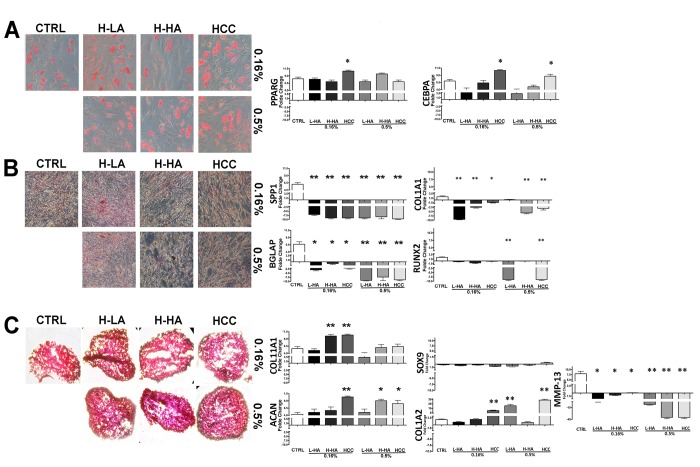
Adipocyte (**A**), osteocyte (**B**), and chondrocyte (**C**) differentiation of MSCs treated with a different HA solution. On the left, there are representative images of Oil Red Oil (**A**), Alizarin Red S (**B**), and Safranin O (**C**) staining for every experimental condition. On the right, quantitative RT-PCR analysis of several differentiation markers. The mRNA levels were normalized to GAPDH mRNA expression, which was selected as an internal control. Histograms show expression levels in the different conditions. Data are expressed as arbitrary units with standard error (± SD, n = 3, *p < 0.05). For each gene in every experimental condition, the expression level in not-differentiated samples is set as the baseline (one value). Up- or down-regulation of genes in differentiated samples is shown as columns above or below the control baselines, respectively.

We evaluated how HA mixtures could affect stemness and multipotentiality of MSCs. To this end, we carried out a CFU assay on the MSC cultures to test their clonogenicity (i.e., their ability to expand at a single-cell level), which is a fundamental feature of self-renewing stem cells. We also evaluated if the tripotential differentiation capacity of MSCs was influenced by HA formulations. Adipo-chondro-osteo differentiation in the presence of each of the tested compounds allowed us to analyze this aspect [13]. The number of MSC clones was reduced after incubation with all the analyzed HA mixtures (Fig. 3). The reduction in CFU clones that we observed prompted us to evaluate if this decrement affected, in the same manner, all the different progenitors or if there was a bias toward a specific lineage-committed clone. MSCs, which we cultivated with the different HA formulations, were then incubated in osteogenic, adipogenic, and chondrogenic differentiating media. These three differentiation pathways were preserved in the presence of the HA mixtures as detected by Alizarin Red S, Oil Red O, and Alcian Blue stainings, respectively (Fig. 4A-B-C). To get further insights on the differentiation process, we analyzed by qRT-PCR the expression levels of several differentiation markers. The mRNA levels of PPAR-gamma (PPARG) and CEBP-alpha (CEBPA), two markers of adipogenesis, were upregulated in the presence of 0.16% HCC compared with the control differentiation cultures. In the other experimental conditions, no significant differences were observed, or in some cases, we evidenced a decline in CEBP-alpha levels (Fig. 4A).

Four markers of osteogenesis (SPP1, BGLAP, COL1A1, RUNX2) were significantly downregulated in all tested experimental conditions compared with controls (Fig. 4B).

A strong increase of COL11A1, a marker of chondrogenesis, was detected in 0.16% HCC and in cells incubated with 0.16% H-HA. Aggrecan (ACAN), another marker of chondrocyte differentiation, showed an increase in 0.16%, 0.5% HCC, and 0.5% H-HA (Fig. 4C). We also analyzed Collagen 2 (COL1A2), SOX9 and MMP-13 to further confirm effects HA complexes on chondrogenis. COL1A2 was upregulated in HCC (0.16% - 0.5%) and L-HA (0.5%). MMP13 showed a decline in all the tested experimental conditions. We barely detected SOX9 and this did not allow to perform quantification (Fig. 4C).

## DISCUSSION

Non-clonal stromal cultures containing a variable amount of MSCs can be easily isolated from a small aspirate of bone marrow and expanded *in vitro.* As such, these cultures are currently used as a source of putative MSCs for therapeutic purposes. Nowadays, dozens of clinical trials aim to treat a number of pathologies, primarily immune system-related diseases, with MSCs [2]. Nevertheless, besides some successes, specifically in the treatment of immunological diseases, MSC therapies have experienced many failures. There are some issues to be analyzed that may improve the success rate of MSC therapy. Donor variance, immunogenicity, and cryopreservation are factors that can impair the effectiveness of MSC cellular therapy [14]. In recent years, another issue has come to the attention of clinicians: expansion differences. Some scientists extensively expanded *in vitro* MSCs for clinical purposes while in other trials MSCs were grown for a limited number of passages *in vitro.* These different procedures may impair MSC functions since *in vitro* cultivation triggers replicative senescence. In some cases, the latter approach appears unavoidable since clinicians require a huge number of cells. The presence of senescent cells in a batch of MSCs that has to be delivered to patients may greatly affect its therapeutic potential because they secreted several factors that may induce senescence of healthy neighbor cells, thus affecting the entire cell batch. This is a typical “dog chasing its tail” case, and most efforts are dedicated to delaying the onset of replicative senescence. To this end, several studies have demonstrated that *in vitro* cultivation procedures should mimic the physiological environment in order to obtain functional cells. In this setting, we focused our attention on HA, which is a key component of many extracellular matrixes, including those forming the stem cell environment.

There are several HA-derived products that can be used for medical purposes. Some HA derivatives contain crosslinked molecules in order to reduce rapid degradation of linear HA in biological environments [15]. Unwanted side effects have been described for HA products presenting a high crosslinking degree and residues of chemical agents that may not be well tolerated by human tissues. Here, we used a new HA-based formulation containing high and low molecular weight HA molecules that contain only natural, identical HA and present, in physiological conditions, hybrid cooperative complexes that optimized through a thermal treatment, during their preparations, the cooperative hydrogen bonding for an interpenetrated network of high and low hyaluronan [15]. This allowed a higher stability than the linear H-HA without chemical modification [7]. In addition, the complexes seemed, in our previous study, to act as slow release systems of the L-HA and H-HA, thus prolonging the effect that is also mediated by specific receptors (e.g., CD44, Rhamm, etc.) [4,5]. The effect of HA on cell physiology is pleiotropic and depends on several factors: the size of HA chains, type of crosslinks, origin of HA, concentration, type of delivery, and target cells [4,16]. In this scenario, comparison of the biological effects of our HCCs with the previous investigations is not a trivial task. We then compared HCCs on the *in vitro* biology of MSCs with those obtained by treating cells with both single compounds as the controls (H-HA and L-HA) to have an internal reference to our findings.

Our results showed that HCC complexes induced a decline in the percentage of cycling cells and an increment of resting cells. These cells were in quiescence rather than in senescence as evidenced by experimental data. The onset of quiescence is in agreement with the reduction in the number of CFU clones that we detected when MSCs were grown in the presence of HCC. Of note, long treatment with 0.16% HCC complexes reduced significantly senescence compared to the control cultures. This occurred without any alteration of the cell cycle profile, cytotoxicity, or apoptosis processes. In agreement with our study, a role for HA in the regulation of senescence has been observed in other systems. Li and co-workers evidenced that replicative senescent fibroblasts have an extracellular matrix with poor HA deposits since they express low levels of hyaluronan synthases (HAS) [17]. Others showed that leukemic cells might survive chemotherapy and escape senescence by increasing the HA synthesis [18].

HCC preserved the adipo-osteo-chondro differentiation as evidenced by histochemical staining. Nevertheless, RT-PCR analysis of differentiation markers indicated that adipogenic and chondrogenic differentiation was increased, while osteogenesis was impaired. Differentiation markers are indicators of progression into a definitively differentiated state. Changes in their expression may be useful to indicate either a specific differentiation phase or advancement toward the final maturation step. On this basis, our data might suggest that HCC complexes accelerated adipogenesis and chondrogenesis while they might negatively affect osteogenesis. This result is in good agreement with studies evidencing the influence of HAs on the *in vitro* chondrogenesis of MSCs [19–21].

Also, H-HA and L-HA complexes preserved the differentiation potential of MSCs, but expression profiles of differentiation markers suggest that the maturation process is delayed and/or impaired. This is at odds with other findings. For example, Zou and co-workers reported that HA might improve osteogenesis of porcine MSCs [22]. Nevertheless, size, concentrations, and time of exposures were different from those we employed. Others evidenced that HA enhances proliferation and differentiation potential of human amniotic MSCs [23]. Also, in this case, experimental conditions were different from those we adopted. Authors used a 300 kDa HA at concentrations lower than those we analyzed. It should be reminded that molecular size, concentration, and crosslinks are discriminating factors in HA interaction with cells [16]. In general, these results suggest that HA-based clinical protocols must carefully consider all the variables that can affect HA functions.

## CONCLUSIONS

Studies are in progress to shed additional light on the molecular events underlying the delay of MSC senescence induced by tested HA formulations.

Our preliminary data indicate that the innovative HCC formulation could pave the way for preparation of new biomaterials by providing an appropriate microenvironment for MSC growth, by preserving their differentiation potential and by delaying unwanted senescence phenomena.

## MATERIALS AND METHODS

### Production of hybrid cooperative complexes

HCCs = Hybrid cooperative complexes, obtained through the patented NAHYCO technology [15], are commercialized by IBSA. This technology is based on thermal procedures for the formation of hybrid cooperative complexes of hyaluronic acid, starting from an initial mixture of an equal amount (ratio 1:1) of H-HA (Mw = 1200±100 kDa Mw/Mn = 1.4) and L-HA (Mw = 100±10kDa, Mw/Mn = 1.4). The experimental concentration used was 32g/L: 32 mg H-HA + 32 mg L-HA in 2 mL volume, provided in prefilled syringes.

In our experiments, we used H-HA = pharmaceutical grade, highly purified linear hyaluronan of 1200 ±100 kDa MW (Altergon Italia, Italy) and L-HA = pharmaceutical grade highly purified linear hyaluronan of 100 ± 10 kDa MW (Altergon Italia, Italy).

### Human MSC cultures

After obtaining bone marrow from healthy donors who had provided their informed consent, we separated cells on a Ficoll density gradient (GE Healthcare, Italy) and collected and washed the mononuclear cell fraction in phosphate-buffered saline (PBS). We seeded 1–2.5 × 10^5^ cells/cm^2^ in modified Eagle’s medium (alpha-MEM) containing 10% fetal bovine serum (FBS) and basic fibroblast growth factor (bFGF). After 72 hr, we discarded non-adherent cells and cultivated adherent ones to confluency. We then further propagated cells for the assays reported below. Cells were used at passage 2 or 3.

### Treatment with hyaluronan complexes

We prepared L-HA, H-HA and HCC mixtures and mixed them with culture media obtaining low viscous solutions. The final concentration of each hyaluronan mixture in the viscous solution was either 0.16% or 0.5%. We seeded 5 × 10^3^ cells/cm^2^ in alpha-MEM containing 10%FBS and bFGF. After 24 hr, medium was replaced with the above indicated viscous solutions and cells were incubated for different time points.

### Cell cycle analysis and immunodetection

For cell cycle analysis and immunostaining, cells were collected and fixed in 70% ethanol followed by PBS washes, and finally, they were dissolved in a hypotonic buffer containing propidium iodide. Samples were acquired on a Guava EasyCyte flow cytometer (Merck Millipore, Italy) and analyzed with a standard procedure using EasyCyte software. We used primary antibodies to detect Ki67 (sc23900, SantaCruz Biotech, CA, USA) and pRPS6 (MC27, Millipore, Italy). Cells were then incubated with corresponding secondary antibodies that were FITC or TRITC conjugated. For each sample, 5,000 cells were evaluated on the Guava instrument. For cell cycle analysis, cells were dissolved in a hypotonic buffer containing propidium iodide. Samples were acquired on a Guava EasyCyte flow cytometer (Merck Millipore, Milano, Italy) and analyzed with a standard procedure using EasyCyte software.

### *In vitro* osteogenic differentiation and Alizarin Red S staining

Osteogenic differentiation was performed by culturing the MSCs with DMEM medium (EuroClone, Pero, Italy) supplemented with 10% FBS (EuroClone, Italy), 0.05mM ascorbic acid (Sigma-Aldrich, MO, USA), 10mM β-glycerophosphate (Sigma-Aldrich, Saint Louis, MO, USA), and 100nM dexamethasone (Sigma-Aldrich, MO, USA) for 21 days, with changes of medium every 3 days. Differentiation was performed either in presence or in absence of the above indicated hyaluronan complexes. Cultures were stained with Alizarin Red S (Sigma-Aldrich, MO, USA) to visualize calcium sediments that were acquired by microscope.

### *In vitro* adipocyte differentiation and Oil Red Oil staining

MSCs were seeded at a density of 1.5x10^4^ in six-well plates and grown in a standard DMEM medium. At 70-80% confluence, the medium was replaced with an adipogenic induction medium composed of high-glucose DMEM (EuroClone, Italy) supplemented with 10% FBS, 1 mM dexamethasone (Sigma-Aldrich, MO, USA), 10 µg/mL insulin (Sigma-Aldrich, MO, USA), 0.5mM 3-isobutyl-1-methylxanthine (Sigma-Aldrich, Saint Louis, MO, USA), and 200 µM indomethacin (Sigma-Aldrich, Saint Louis, MO, USA). Cells were cultured in this medium for 21 days.Differentiation was performed either in presence or in absence of the above indicated hyaluronan complexes.

Adipogenic differentiation was confirmed on day 21 using an Oil Red O stain (Sigma-Aldrich, Saint Louis, MO, USA) as an indicator of intracellular lipid accumulation. Briefly, cells were washed twice in PBS, fixed with 4% formaldehyde for 10 min at room temperature, rinsed once with 3% isopropanol, and stained with Oil Red O staining solution. Then, cells were rinsed with water and photographed under the microscope.

### *In vitro* chondrogenic differentiation and Safranin O staining

Chondrogenic differentiation was performed in 3D culture. Briefly, 1x10^5^ cells were seeded as a pellet in 96 round bottom multi-wells and cultured in achondrogenic medium composed of DMEM, 1% FBS, 50 nM ascorbate-2-phosphate (Sigma-Aldrich, MO, USA), 0.1 mM dexamethasone (Sigma-Aldrich, Saint Louis, MO, USA), and 10 ng/mL human transforming growth factor (hTGF)-β1 (Preprotech, UK). The medium was replaced every 3 days. Differentiation was performed either in presence or in absence of the above indicated hyaluronan complexes.

After 21 days, Safranin O staining was performed to detect glycosaminoglycan formation on the cell surfaces, (Sigma-Aldrich, MO, USA) In short, cell pellets were fixed in cold (4°C) acetone:methanol solution for 60 min and then included in cryostat embedding medium (Bioptica, Italy) for cryosectioning. Pellet sections were incubated at room temperature in 1% Safranin O solution for 30 min followed by three rinses in 3% acetic acid for 2 min each. After rinsing in deionized water for 2 min, the surfaces were allowed to dry for imaging.

### Colony Forming Units assay (CFU)

MSC cultures were obtained as described above. Cultures were expanded to 70–80% confluence. On these cells, we carried out a CFU assay. Briefly, we plated 1,000 cells in 10 cm culture dish and incubated for 14 days in a growth medium. Subsequently, the medium was discarded, and colonies were fixed with 100% methanol for 10 minutes at -20°C. Colonies were then stained 0.01% (w/v) crystal violet (Sigma-Aldrich, MO, USA) in 25% methanol in PBS for 30–60 min. For every experimental condition, we counted the number of colonies in culture dishes at light microscope.

### Annexin-V assay

Apoptotic cells were detected using Nexin V kit (Millipore, Italy) on a Guava EasyCyte flow cytometer, following the manufacturer’s instructions. The kit utilizes two separate dyes (Annexin V and 7AAD) to identify a broad spectrum of apoptotic and non-apoptotic cells. Annexin V (red) binds to phosphatidylserine on the external membrane of apoptotic cells, while 7AAD (blue) permeates and stains DNA of late-stage apoptotic and dead cells. Staining allows the identification of three cell populations: non-apoptotic cells (Annexin V- and 7AAD-); early apoptotic cells (annexin V+ and 7AAD-); and late-apoptotic or dead cells (Annexin V+ and 7AAD+). In our experimental conditions, early and late apoptotic cells were grouped.

### *In situ* senescence-associated beta-galactosidase assay

Cells were fixed using a solution of 2% formaldehyde and 0.2% glutaraldehyde. After this, cells were washed with PBS and then incubated at 37°C for at least 2 hr with a staining solution (citric acid/phosphate buffer (pH 6), K_4_Fe(CN)_6_, K_3_Fe(CN)_6_, NaCl, MgCl_2_, and X-Gal). The percentage of senescent cells was calculated by the number of blue, b-galactosidase-positive cells out of at least 500 cells in different microscope fields as already reported*.*

### RNA extraction, RT-PCR, and real-time PCR

We extracted total RNA from cell cultures using Omnizol (EuroClone, Italy), according to the manufacturer’s protocol, and measured mRNA levels by RT-PCR amplification.

We used sequences of mRNAs from the Nucleotide Data Bank (National Center for Biotechnology Information, MD, USA) to design primer pairs for real-time RT-PCR reactions (Primer Express, Applied Biosystems, Italy); primer sequences are available upon request. We used appropriate regions of HPRT and/or GAPDH cDNA as controls and ran real-time PCR assays on an Opticon 4 machine (MJ Research, MA, USA). We carried out reactions according to the manufacturer’s instructions using a SYBR green PCR master mix and used the 2^-ΔΔCT^ method as a relative quantification strategy for quantitative real-time PCR data analysis.

### Statistical analysis

We evaluated statistical significance using analysis of variance, followed by Student’s t and Bonferroni’s tests. For data with continuous outcomes, we used mixed-model variance analysis and, in any case, analyzed all data with GraphPad Prism version 5.01 (GraphPad, La Jolla, CA, USA).
